# Characterization of Natural Dyes and Traditional Korean Silk Fabric by Surface Analytical Techniques

**DOI:** 10.3390/ma6052007

**Published:** 2013-05-15

**Authors:** Jihye Lee, Min Hwa Kang, Kang-Bong Lee, Yeonhee Lee

**Affiliations:** 1 Advanced Analysis Center, Korea Institute of Science and Technology, Seoul 136-791, Korea; E-Mails: stern07@lycos.co.kr (J.L.); kmin1127@gmail.com (M.H.K.); leekb@kist.re.kr (K.-B.L.); 2 Department of Nano and Bio Surface Science, University of Science and Technology, Daejeon 305-350, Korea

**Keywords:** natural dyes, traditional silk fabric, mordants, TOF-SIMS, XPS, FTIR, surface techniques

## Abstract

Time-of-flight secondary ion mass spectrometry (TOF-SIMS) and X-ray photoelectron spectroscopy (XPS) are well established surface techniques that provide both elemental and organic information from several monolayers of a sample surface, while also allowing depth profiling or image mapping to be carried out. The static TOF-SIMS with improved performances has expanded the application of TOF-SIMS to the study of a variety of organic, polymeric and biological materials. In this work, TOF-SIMS, XPS and Fourier Transform Infrared (FTIR) measurements were used to characterize commercial natural dyes and traditional silk fabric dyed with plant extracts dyes avoiding the time-consuming and destructive extraction procedures necessary for the spectrophotometric and chromatographic methods previously used. Silk textiles dyed with plant extracts were then analyzed for chemical and functional group identification of their dye components and mordants. TOF-SIMS spectra for the dyed silk fabric showed element ions from metallic mordants, specific fragment ions and molecular ions from plant-extracted dyes. The results of TOF-SIMS, XPS and FTIR are very useful as a reference database for comparison with data about traditional Korean silk fabric and to provide an understanding of traditional dyeing materials. Therefore, this study shows that surface techniques are useful for micro-destructive analysis of plant-extracted dyes and Korean dyed silk fabric.

## 1. Introduction

Organic dyes are very significant materials in archaeological remains. Investigation of the coloring materials used on ancient textiles provides a historical window to understanding the surrounding environment, the living style and the technological skill in ancient times [1,2,3,4]. However, dyes have complicated chemical composition and can co-exist with other chromophores and contaminants, so that the identification of organic dyes in old textiles is a challenging task. There are four major barriers to characterizing organic dyes: (1) the relatively small quantity of samples available; (2) the low concentration of chromophores in the textiles; (3) the presence of possible degradation products; and (4) the lack of information on the original recipes [5,6,7].

For last two decades, chemical research in the field of the analytical separation and spectrometric identification of organic dyes has been performed and studies have presented a new chromatographic scheme for the identification of certain dyes [8]. The identification of organic dyes in various types of textiles was conducted using chromatographic techniques, such as liquid chromatography mass spectrometry, gas chromatography mass spectrometry, high performance liquid chromatography and thin-layer chromatography [9,10,11,12]. The extraction of dyes from textiles is a critical process in this type of analytical techniques due to the small amount of sample available. Using HPLC analysis, a successful new mild extraction method was established for the identification of dyes in historical remains [13,14].

Recently, researchers have focused great attention on analytical techniques based on direct mass spectrometry, such as direct exposure mass spectrometry (DE-MS), laser desorption/ionization mass spectrometry (LDI-MS), matrix assisted laser desorption/ionization mass spectrometry (MALDI-MS) and time-of-flight secondary ion mass spectrometry (TOF-SIMS) [15,16,17,18]. Compared to wet chemical preparation, these techniques involve minimum sample requirement, no sample pretreatment, less contamination and reduced samples loss. Furthermore, the analysis times are very short and the sensitivities are suitable for identifying organic dyes in textiles. Lee *et al.* used TOF-SIMS to study natural dyes and ancient textiles from Korea [17]. Indigo was successfully identified by TOF-SIMS for two ancient textiles from 16th and 17th-century Korea.

In this study, comparing to the conventional FTIR technique, to avoid extraction and to use minimum sample amount, surface analytical techniques, such as TOF-SIMS and X-ray photoelectron spectroscopy (XPS), were used to detect natural dyes in silk fabric. TOF-SIMS has been found to be an ideally suited analytical technique to characterize surface compositions and structures, because it provides elemental and molecular information about the surface layers with a high degree of sensitivity, mass resolution and special resolution [19]. XPS occupies a unique position in the field of archaeological analysis, because it permits examination of an extensive array of elements from the surfaces of artifacts and because it offers sensitivity to elemental oxidation states [20]. FTIR spectroscopy has been applied to the characterization of specific dyes in textiles; however, signals from the matrix severely interfere with the identification of the coloring materials [21]. Therefore, a complementary analysis of various natural dyes and silk fabric dyed with plant extracts was performed by TOF-SIMS, XPS and FTIR.

## 2. Experimental Section

### 2.1. Materials 

The commercial natural dyes, such as curcumin, indigo and quercetin, were purchased from the Tokyo Chemical Industry (TCI) Co., LTD. Tokyo, Japan. Both brazilin and shikonin were supplied by the Sigma-Aldrich Chemical Company, Inc., St. Louis, MO, USA. Natural dyes were also extracted from four plants. In this case, turmeric and sappanwood plants were imported from India and the Philippines, whilst gromwell and indigo plants were all readily available in Korea.

#### 2.1.1. Dyeing Using Plant Extracts

The majority of the natural plant dyes were extracted in water either by soaking at room temperature or by heating. Turmeric (20 g) was placed in 1 L of methanol for 7 days. Silk fabric (10 cm × 10 cm) was soaked in 2.5 mL of turmeric extract diluted with 300 mL of water at 40 °C for 60 min. Indigo plant was soaked in water for 4 days. Then, Ca(OH)_2_ was added to the solution of 250 mL, and the resulting precipitate was filtered and dried. Silk fabric of 10 cm × 10 cm has been dyed in 1.7 L of water mixed with 100 g of precipitated dye. For zinc mordanting, silk fabric was treated in 1.7 L of water containing 100 g of dye, 3.3 g of zinc powder and 7 g of sodium hydroxide at room temperature for 60 min. Sappanwood (200 g) was soaked in 2 L of water and solution was heated. Silk fabric (10 cm × 10 cm) was dyed in dye solution for 30 min. After dyeing, the dyed fabric was washed in running water and dried at room temperature. Silk fabric was treated with different metal salts. In the case of Al mordant, silk fabric was treated in 400 mL of dye solution containing 2 g of alum at 40 °C for 30 min. For Fe mordant, silk fabric was dyed in 200 mL of dye solution for 30 min and then treated in 200 mL of water containing 3 g of iron sulfate (II) for 15 min. Sn mordanting was carried out in 200 mL of water with 0.08 g of tin chloride (IV) for 15 min and dyed in dye solution for 30 min. In the case of Cu mordant, silk fabric was dyed in dye solution at 40 °C for 30 min and washed. After pre-dyeing, fabric was treated in copper sulfate for 15 min and rinsed with water. Gromwell (50 g) was soaked in water at 40 °C for 3 h and peeled slightly. Then, colorant was extracted kneading macerated gromwell in 1 L of water at 60 °C for 20 min. After filtering the solution, silk fabric (10 cm × 10 cm) was dyed in dye solution at 60 °C for 20 min two times. Dyed silk fabric was rinsed with water and dried at room temperature.

#### 2.1.2. Dyeing Using Commercial Dyes

Silk fabric (10 cm × 10 cm) was dyed in 300 mL of water containing 1 g of curcumin powder at 60 °C for 30 min and dyeing process was repeated two times. In order to use indigo for dyeing fabrics, it must be transformed to the soluble form of indigo, known as leuco-indigo. Five grams of sodium hydroxide was dissolved in 200 mL of distilled water and added 5 g of sodium hydrosulfite. After removing insoluble material, silk fabric (10 cm × 10 cm) was dyed in 55 °C for 30 min and rinsed in cold water. Silk fabric (10 cm × 10 cm) was dyed in 300 mL of water containing 1 g of brazilin, shikonin or quercetin powder at 40 °C for 30 min and dyeing process was repeated two times. A detailed description of dyeing methods is presented elsewhere [22]. Table 1 shows the empirical formula, molecular weight and chemical structures of the major components of plant dyes selected for this work. Table 2 shows various colors and colorimetric parameter of textiles dyed with commercial dyes and natural dyes extracted from plants.

**Table 1:** Details of the natural dyes extracted from plants.

Major dye component MW Color	Name of plant (English)	Chemical structure
Curcumin C_21_H_20_O_6_ 368.38 Yellow	*Curcuma longa L.* (Turmeric)	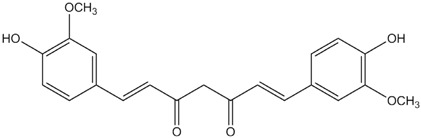
Indigo C_16_H_10_N_2_O_2_ 262.26 Blue	*Indigofera tinctoria* (Indigo)	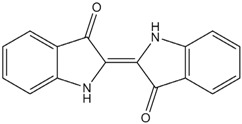
Brazilin C_16_H_14_O_5_ 286.27 Red	*Caesalpinia sappan L.* (Sappanwood)	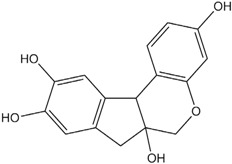
Shikonin C_16_H_16_O_5_ 288.29 Purple	*Lithospermum erythrorhizon S. et Z* (Gromwell)	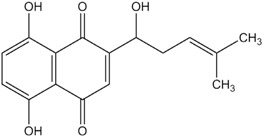
Quercetin C_15_H_10_O_7_ 302.24 Yellow	*Sophora japonica L.* (Japanese pagoda tree)	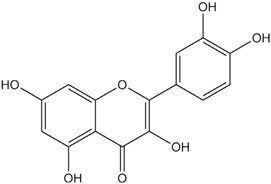

**Table 2:** Various colors and colorimetric data of silk fabric dyed with commercial dyes, plant extracts dyes and plant extracts dyes with mordants. (L*,a*,b* is CIELAB color scale)

Commercial dyes			Natural dyes from plant extracts			Plant extracts dyes + Mordants		
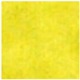 Curcumin	L*	87.30	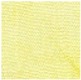 Turmeric	L*	85.83	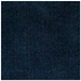 Indigo (Zn)	L*	31.97
	a*	−12.88		a*	−8.09		a*	0.39
	b*	69.33		b*	48.63		b*	−18.11
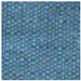 Indigo	L*	51.82	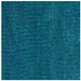 Indigo	L*	49.78	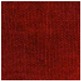 Sappanwood (Al)	L*	37.20
	a*	−3.38		a*	−7.27		a*	42.10
	b*	−19.96		b*	−20.55		b*	22.38
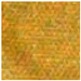 Brazilin	L*	69.49	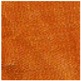 Sappanwood	L*	58.92	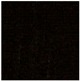 Sappanwood (Fe)	L*	19.81
	a*	2.59		a*	21.05		a*	2.49
	b*	29.67		b*	42.79		b*	1.03
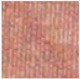 Shikonin	L*	67.77	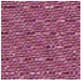 Gromwell	L*	−10.30	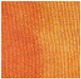 Sappanwood (Sn)	L*	61.51
	a*	18.67		a*	19.66		a*	28.71
	b*	4.46		b*	−18.12		b*	27.81
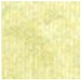 Quercetin	L*	76.62	–			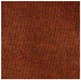 Sappanwood (Cu)	L*	34.77
	a*	−3.52					a*	21.35
	b*	13.40					b*	11.95

### 2.2. Instrumental Evaluation 

Time-of-Flight Secondary Ion Mass Spectrometry. Positive and negative ion TOF-SIMS analyses were carried out on an ION-TOF GmbH (Münster, Germany). A TOF-SIMS 5 system was equipped with a Bi^+^ primary ion beam source. A Bi^+^ liquid metal ion source (LMIS) was employed for mass data acquisition. Such cluster ions provide better secondary ion yields than those available from the more conventional Ga^+^ source. The analysis source was a pulsed 25-keV Bi^+^ beam that bombarded the surface at an incident angle of 45° to the surface normal. The target current was maintained at a pulsed current of 1 pA with a raster size of 100 μm × 100 μm. A cycle time of 100 μs was employed for mass data acquisition. These conditions resulted in Bi^+^ ion doses that were well below the static SIMS limit of 10^13^ ions/cm^2^.

Both positive and negative secondary ions were extracted from the sample and accelerated using an ion lens operating at 2 kV into a time-of-flight (TOF) mass analyzer. The TOF uses a reflectron to reduce line widths and has a total flight path of 2 m. The secondary ion beam is focused on the microchannel plate with a post-acceleration energy of 20 kV. A low-energy electron flood gun was used for charge compensation of all silk textile samples.

X-ray Photoelectron Spectroscopy. X-ray photoelectron spectroscopy (XPS) was used to examine the chemical structure on the surface of natural dyes on silk fabric using a PHI-5000 VersaProbe instrument (ULVAC-PHI, Kanagawa, Japan) with a base pressure of 6.7 × 10^−8^ Pa. The dyed silk textiles were analyzed by measuring the C1s, N1s and O1s and the metal element spectra in the XPS. The XPS analysis was conducted with monochromatic Al Kα radiation (1486.6 eV) at a power of 25 W. The instrument was calibrated to the 4f_7/2_ peak of gold at E_b_ = 84.03 eV with a full width at half maximum (FWHM) of 0.71 eV and to the copper 2p_3/2_ peak at E_b_ = 932.66 eV with a FWHM of 0.91 eV. Charging of the dyed silk textile, as a result of photoemission, was corrected by setting the energy of the main hydrocarbon component of the C1s spectrum to 284.6 eV for dyed textiles. A pass energy of 23.5 eV was utilized for all narrow spectra. The spot size of the X-ray beam was 100 μm × 100 μm in each case. The sample was exposed to X-rays for several minutes to minimize the X-ray beam damage.

Fourier Transform Infrared Spectroscopy. Attenuated Total Reflection Fourier Transform Infrared (ATR-FTIR) measurements were carried out with an Infinity Gold FT-IR Series (ThermoMattson, Waltham, MA, USA) system equipped with an IR microscope (SpectraTech, Inspect IR plus, Franklin Lakes, NJ, USA) with a mercury cadmium telluride detector. One hundred twenty eight scans were collected with a resolution of 4 cm^−1^, and the frequency range was measured between 600 and 4000 cm^−1^ of the mid-IR region. Dyed silk fabric were mounted in the sample holder and no additional sample preparation was done. Spectrum of the undyed silk textile was recorded and this served as the blank throughout the experiment. Several spectra of the undyed silk textile were done to check for the reproducibility.

Color measurements. Color measurements of the dyed silk fabric were performed directly using a spectrophotometer (Minolta Chromameter, CM-5).

## 3. Results and Discussion

### 3.1. FT-IR Measurement 

**Figure 1:**
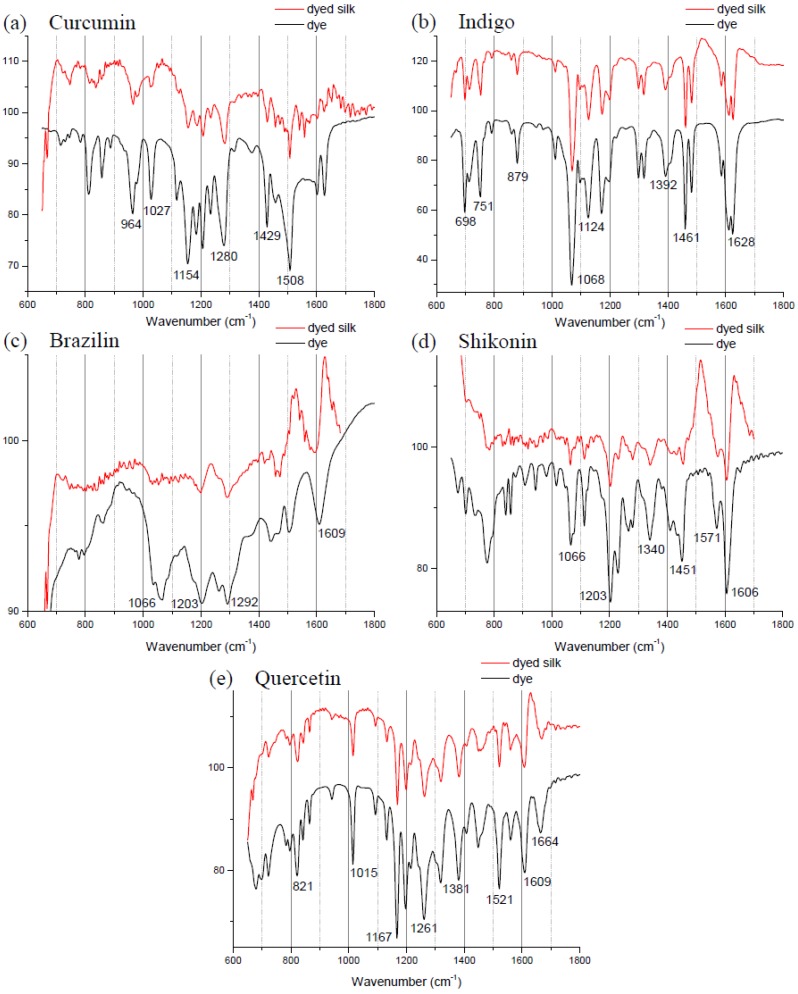
Fourier transform infrared spectra of five commercial dyes and the dyed silk fabric between 600 and 1800 cm^−1^: (**a**) curcumin; (**b**) indigo; (**c**) brazilin; (**d**) shikonin; and (**e**) quercetin.

Table 1 shows the empirical formulas, molecular weights and chemical structures of the major components of the plant dyes selected for this work; these include curcumin, indigo, brazilin, shikonin and quercetin. To establish the FTIR technique for dye identification, commercial dyes and the dyed silk fabric were prepared. Figure 1 compares the FTIR spectra of curcumin, indigo, brazilin, shikonin and quercetin-dyed silk fabric obtained by subtraction of undyed silk with one obtained from the commercial dyes. Undyed silk textile was used to obtain the reference spectrum. No dye component could be detected in the FTIR spectra unless spectral subtractions were conducted. After spectral subtraction, it was easily possible to identify the dye component in all of the dyed silk fabric.

The spectrum of indigo is shown with a specific peak at 2360 cm^−1^ due to the N–H stretching, a peak at 1628 cm^−1^ that is because of C=O stretching and a peak at 1392 cm^−1^ that is because of N–H bending. Other specific peaks are the aromatic ring C=C vibration at 1461, 1483 and 1585 cm^−1^.

**Figure 2:**
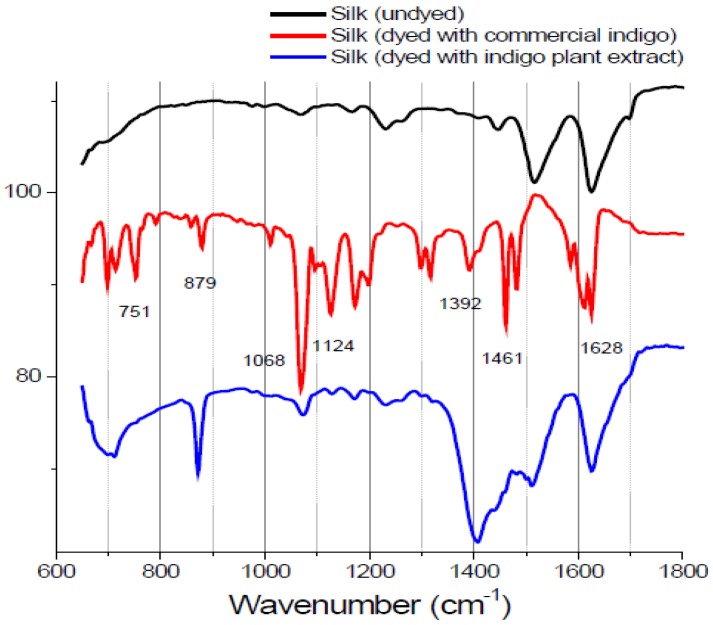
Fourier transform infrared spectra of undyed silk textile with commercial indigo and silk textile dyed with indigo plant extract. The characteristic peaks allow easy identification. The spectra are vertically shifted for sake of clarity.

Minerals readily available from the environment were used in combination with natural dyes to provide a strong bond between the textile and the dye. Color brightness and fastness are improved by the use of mordants in the dyeing process. However, the mordant had no apparent effect on the FTIR spectra. The FTIR spectra of the indigo extract-dyed and zinc-mordanted silk fabric that underwent spectral subtraction of undyed silk from indigo-dyed silk matched those of silk fabric dyed with commercial indigo, with specific peaks at 1628, 1124 and 1068 cm^−1^ (Figure 2). These results, which are expected, because indigo is the main coloring component in indigo plant, support the accuracy of the FTIR technique. However, it was not possible to distinguish individual dye components in silk fabric dyed with the other natural extracts using the FTIR technique. In this study, we prepared dyed silk fabric using the traditional dying process; the amounts of dyes used in this procedure may be below the detection limit of FTIR.

### 3.2. XPS Measurement 

**Figure 3:**
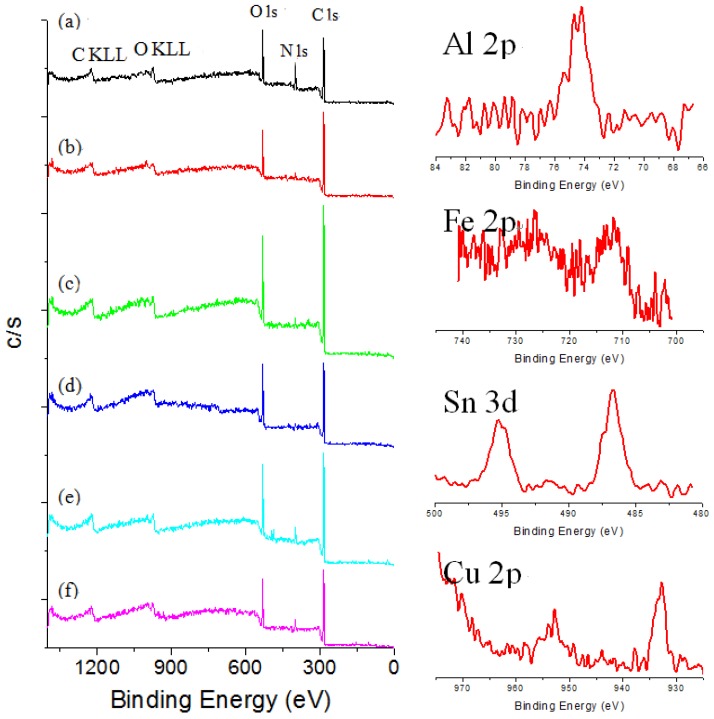
Survey XPS spectra of (**a**) undyed silk and sappanwood-dyed silk textiles; (**b**) without mordant and with various mordants: (**c**) KAl(SO_4_)_2_; (**d**) FeSO_4_; (**e**) SnCl_4_; and (**f**) CuSO_4_.

XPS techniques have rarely been applied to the identification of specific dyes in textiles, because signals due to the textile heavily interfere with the identification of coloring materials. The XPS survey spectra for dyed silk fabric were obtained to determine chemical compositions of silk textiles. C1s and O1s peaks are the main signals in the survey spectra of the silk fabric dyed with curcumin, brazilin, shikonin and quercetin, while C1s, O1s and N1s peaks are the main signals for those dyed with indigo. As can be seen in Figure 3, the survey spectra of sappanwood-dyed silk fabric reveal the presence of aluminum, iron, tin and copper due to the addition of mordants during the dyeing process. The calculated atomic concentrations obtained by XPS for the sappanwood-dyed silk fabric are listed in Table 3. As expected, the surface compositions of these silk textiles are similar. The discrepancy of carbon and oxygen concentrations from the theoretical values was probably caused by hydrocarbon and remaining vapor contamination, the textile surface and the surface degradation induced by X-ray. The amounts of mordants are rather low, so their peaks are not intense in the XPS spectra. However, quantitative results for various mordant elements in dyed silk fabric were obtained by the XPS technique.

**Table 3:** XPS Surface compositions of silk fabric dyed with sappanwood extracts and different mordants.

Sample/Mordant	Elements composition (%)						
	C (%)	O (%)	N (%)	Al (%)	Fe (%)	Sn (%)	Cu (%)
Sappanwood	77.05	18.28	4.66	–	–	–	–
Sappanwood/K,Al(SO_4_)_2_	76.28	21.14	1.53	1.04	–	–	–
Sappanwood/FeSO_4_	74.21	22.77	2.17	–	0.85	–	–
Sappanwood/SnCl_4_	73.18	20.34	6.12	–	–	0.36	–
Sappanwood/CuSO_4_	73.66	20.62	4.63	–	–	–	1.09

**Figure 4:**
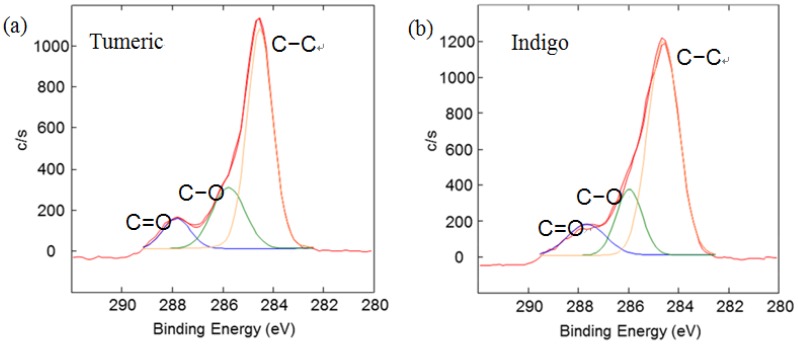

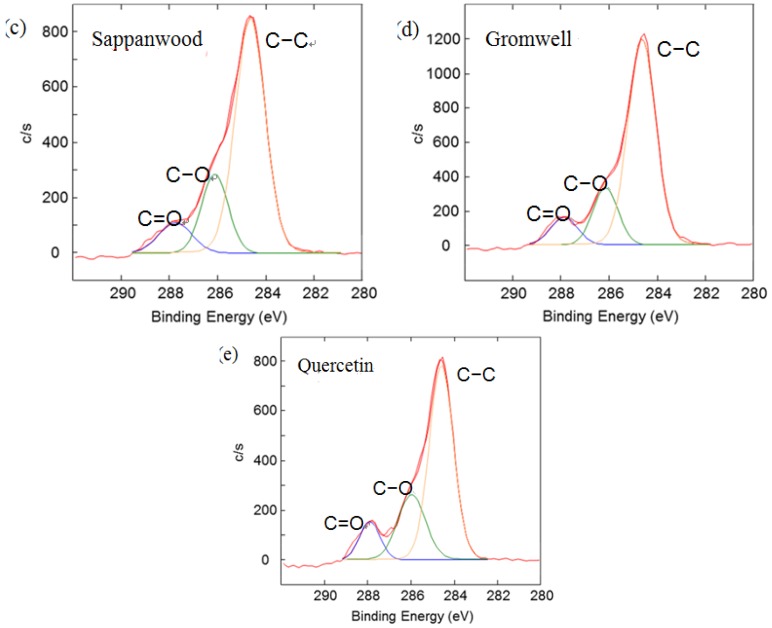
Results of the curve fitting analysis of C1s spectrum measured for silk textiles dyed with plant extracts. (**a**) Turmeric; (**b**) Indigo; (**c**) Sappanwood; (**d**) Gromwell; (**e**) Quercetin.

To offset the surface charging phenomena, all binding energies in the C1s core level spectra were referenced to the C–C peak centered at 284.6 eV. The peak deconvolution of the XPS result was performed by Gaussian-Lorentz mixture function using MULTI-PAK v9.1 software. Figure 4 illustrates C1s peaks of dyed silk fabric resolved using curve fitting. The XPS results for the silk fabric dyed with turmeric, indigo, sappanwood, gromwell and quercetin are listed in Table 4. The table presents the percentages of each carbon functional group presented in the spectra. The peak fitting was performed with a consideration of parameters, such as binding energy, full width at half maximum and Gaussian character. The fitting parameters for the dye components of the dyed silk textile in the C1s spectrum were determined by analyzing the C1s spectrum measured for a sample of commercial dye. Line-shape analysis using peak deconvolution shows that the C1s spectrum for undyed silk textile contains three distinct peaks at 284.6 (–C–C), 286.2 (–C–OH or –C–N) and 288.0 eV (N–C=O). These peaks may be attributed to the bonds present in the silk and to any residual surface contaminants. After the dyeing process with plant extracts, the intensity of C1s increased and C1s spectra showed peaks of oxygen-containing groups at 286.2 (–C–OH or –C–O–C) and 287.6 eV (–C=O) with different intensities. As shown in the chemical structures of Table 1, turmeric and quercetin have more oxygen-containing functional groups, like hydroxyl and carboxyl groups, than gromwell. XPS results also indicated that turmeric and quercetin had more C–OH and C=O bonds than gromwell. The XPS technique provided the quantification results for the surface composition of the dyed silk textiles with various plant extracts and mordants.

**Table 4:** Relative concentration of C1s components for silk fabric dyed with plant extracts.

Sample	Relative chemical bond area Cls (%)		
	C−C (284.6 eV)	C−O C−N (286.2 eV)	C=O (287.7 eV)
Turmeric	67.22	23.28	9.51
Indigo	69.55	18.08	12.37
Sappanwood	69.92	20.32	9.76
Gromwell	73.10	17.73	9.17
Quercetin	65.17	24.26	10.58

### 3.3. TOF-SIMS Measurement

**Figure 5:**
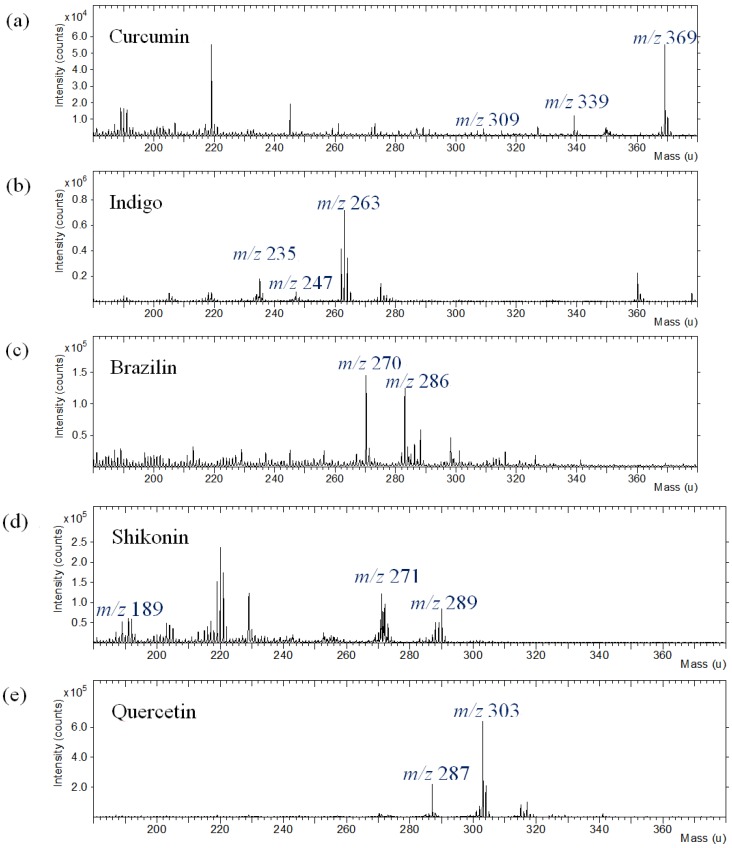
Positive ion TOF-SIMS spectra of commercial natural dyes on the silicon substrate. (**a**) curcumin; (**b**) indigo; (**c**) brazilin; (**d**) shikonin; (**e**) quercetin.

The commercial dyes used in this work were curcumin, indigo, brazilin, shikonin and quercetin. Five commercial natural dyes were purchased from the manufacturer, and their TOF-SIMS spectra were obtained from thin layer films cast from solutions of dyes in solvent on a silicon substate. The TOF-SIMS technique provides molecular structure and composition information from the uppermost layer of the dye components. To characterize the commercial natural dyes, the positive ion TOF-SIMS mass spectra were acquired at the film surface of the dyes. The positive ion mass spectra of the dyes were acquired using a Bi^+^ primary ion beam under static SIMS conditions from a solution-cast dye film exposed to room temperature, as shown in Figure 5.

Interpretation of the spectra will focus on the information they provide for structural characterization. A segment from a typical spectrum of dyes in the mass range of m/z = 0–100 shows that the dominant peaks are C_2_H_3_^+^ (m/z 27.023), C_2_H_5_^+^ (m/z 29.039), C_3_H_5_^+^ (m/z 41.039), C_3_H_7_^+^ (m/z 43.055), C_2_H_3_O^+^ (m/z 43.018), C_4_H_7_^+^ (m/z 55.055), C_3_H_3_O^+^ (m/z 55.018), C_4_H_9_^+^ (m/z 57.070) and C_3_H_5_O^+^ (m/z 57.034). Na^+^ (m/z 22.990) and K^+^ (m/z 38.964) ions are also found in the spectra, as well as Si^+^ (m/z 27.977) from the silicon substrate (not shown in the figure). The other peaks in the lower mass range are due to carbon- and oxygen-containing ions that are found in the positive spectra of nearly all organic materials.

In the higher mass range between m/z 180 and 380, the positive TOF-SIMS spectra of curcumin (Figure 5a) indicate the presence of molecular ions, M+H at m/z 369 and specific fragment ions, *i.e.*, C_10_H_9_O_3_^+^ at m/z 177, C_19_H_17_O_4_^+^ at m/z 309 and C_20_H_19_O_5_^+^ at m/z 339. The positive ion spectrum of indigo shows characteristic ions at m/z 120 (C_7_H_6_NO^+^), 132 (C_8_H_6_NO^+^), 235(C_15_H_11_N_2_O^+^) and 247 (C_16_H_11_N_2_O^+^), as well as molecular ion (M+H) at m/z 263. TOF-SIMS spectra of the other dye compounds studied, such as brazilin, shikonin and quercetin, also show molecular ions at m/z 286 (M^+^), m/z 289 (M+H) and m/z 303 (M+H) and specific fragment ions in the positive ion spectra.

**Figure 6:**
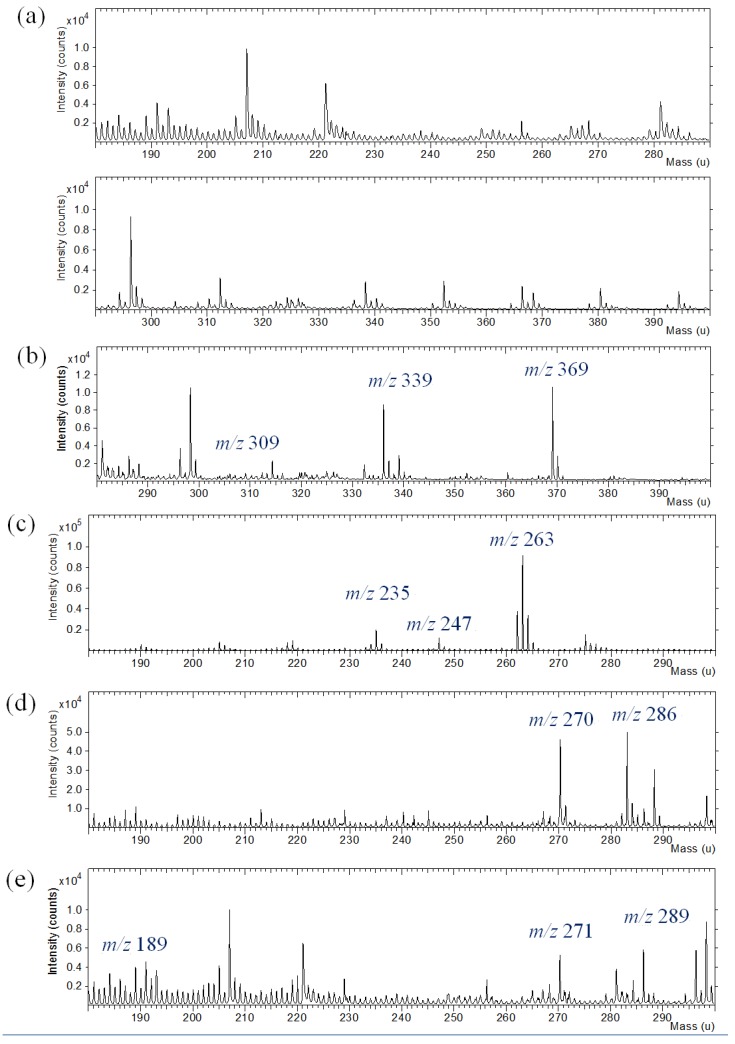

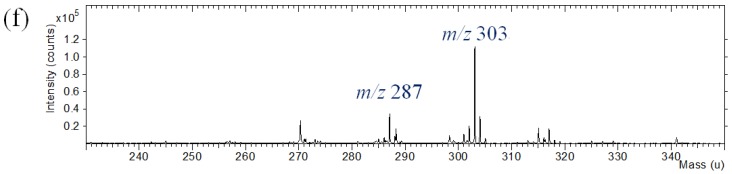
Positive ion TOF-SIMS spectra of silk fabric (**a**) undyed and dyed with commercial natural dyes: (**b**) curcumin; (**c**) indigo; (**d**) brazilin; (**e**) shikonin; (**f**) quercetin.

Silk fabric were dyed with commercial natural dyes. TOF-SIMS analysis of dyed silk textile provided complicated spectra composed of peaks from dyes, silk fabric and contaminants. The TOF-SIMS spectrum of silk textile was also obtained to discriminate the background information from the silk textile. The silk textile gives hydrocarbon ions and nitrogen-containing ions in the low mass range below m/z 200. TOF-SIMS spectra of silk fabric dyed with curcumin, indigo, brazilin, shikonin and quercetin are shown in Figure 6. In the silk fabric dyed with curcumin, indigo, brazilin, shikonin and quercetin, molecular ions (M+H or M^+^) at m/z 369, 263, 286, 289 and 303 were observed with strong intensity. The specific fragment ions from the dye molecules were also identified in the complicated background peaks from the silk textile.

**Figure 7:**
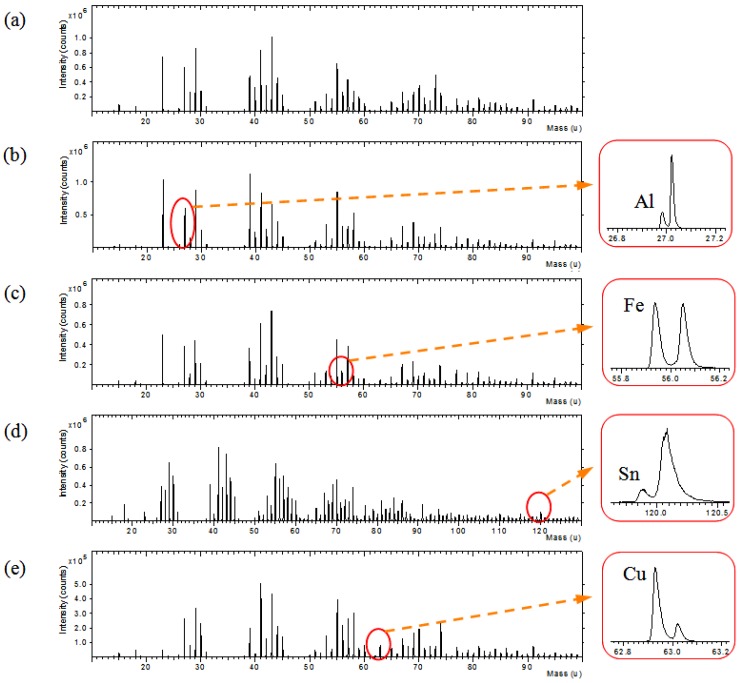
Positive ion TOF-SIMS spectra of sappanwood-dyed silk textiles (**a**) without mordant and with various mordants: (**b**) KAl(SO_4_)_2_; (**c**) FeSO_4_; (**d**) SnCl_4_; and (**e**) CuSO_4_.

Silk textiles were prepared with plant dye extracts and mordants or fixing agents in the dyeing process, because many of natural dyes did not have a strong chemical affinity for the textile fibers. As shown in Figure 7, TOF-SIMS spectra were obtained for the silk textiles dyed with sappanwood extracts and different kinds of mordants. In the low mass range of m/z 0–100, intense element ions from metal mordants were easily observed. Other metal ions in TOF-SIMS spectra were observed at the background level that might come from the contamination. The presence of aluminum, iron, tin and copper ion was due to the addition of mordants during the dyeing process of the silk fabric. High mass resolution TOF-SIMS spectra provided the characteristic ions from most mordant elements and dye molecules, separated with many hydrocarbon peaks. Therefore, TOF-SIMS is a useful technique to investigate natural dyes.

**Figure 8:**
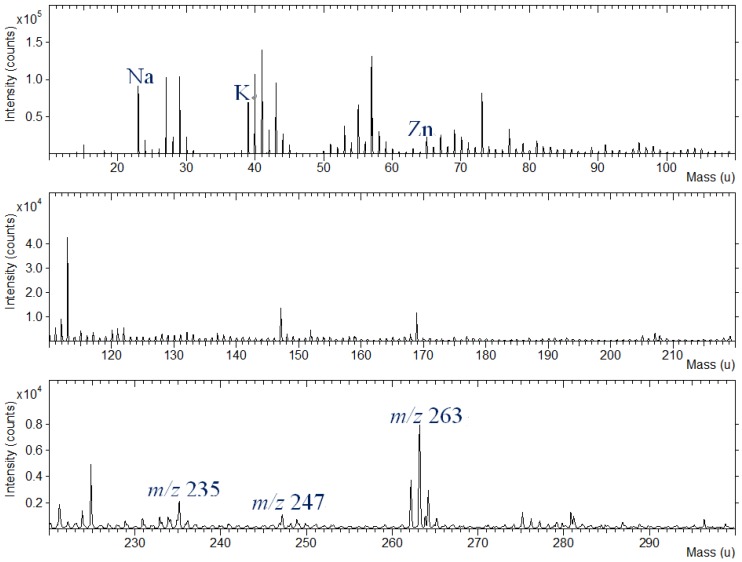
Positive ion TOF-SIMS spectrum of silk textile dyed with indigo plant extract and Zn powder.

Silk fabric were dyed with various dye extracts of plants. Representative TOF-SIMS spectrum of silk textile dyed with indigo in the mass range of m/z 10–300 is shown in Figure 8. As can be seen in the figure, the specific fragment ions are present at m/z 235 and 247; as well, the molecular ion (M+H) of indigo can be observed at m/z 263. The specific zinc peaks from the mordants are also shown in this figure. 

**Figure 9:**
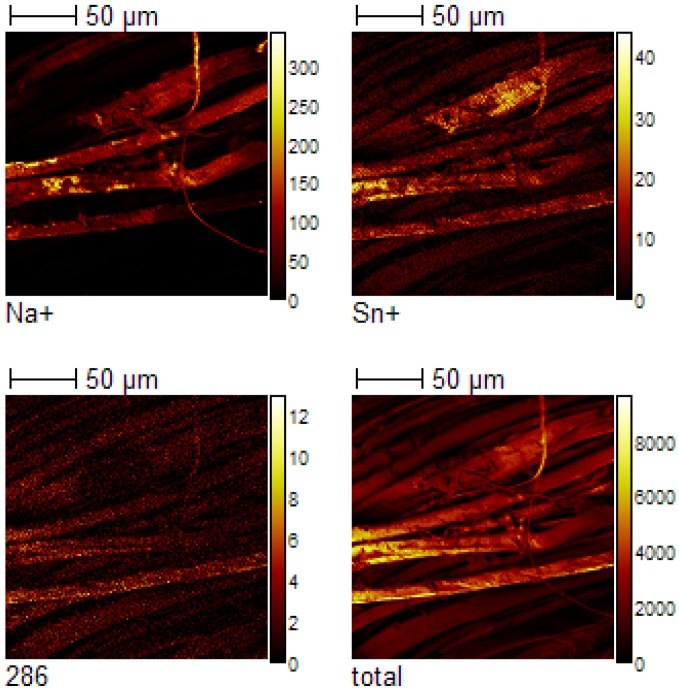
Positive TOF-SIMS images of sappanwood on silk textile: Na(m/z 23), Sn(m/z 120), brazilin(m/z 286) and total ion.

The use of TOF-SIMS to characterize the chemical distribution of dye components on silk fabric was explored. TOF-SIMS imaging data was obtained for the silk textile dyed with sappanwood and SnCl_4_. Figure 9 provides ion images obtained from the positive ion TOF-SIMS spectrum. No clear contrast between the dye distribution, and the silk textile surface is discernible. The TOF-SIMS images show the special distribution of Na^+^, Sn^+^, molecular ion (C_16_H_14_O_5_^+^, 286) and total ions. The distribution of the molecular ion exhibits a uniform distribution over the analyzed area, whereas the sodium and tin appear to be located in small aggregates. The TOF-SIMS image results indicate that most fractions of the silk fibers are covered by the plant extracted dye component during the traditional dyeing process according to the old recipes. However, metallic ion from the mordant agent was not distributed uniformly in the silk fibers. Sodium is not an element included in sappanwood component or the mordant. Sodium usually comes from the contamination of the sample and is easily ionized by the primary ion beam. The area contaminated by sodium was different in TOF-SIMS image map from those of dye component and mordent element.

Therefore, plant extracted dyes on the silk textiles have been identified using the TOF-SIMS technique on a scale far below that which is possible when using the extractive technique. The TOF-SIMS technique provided information on both elements, including isotope discrimination and molecules, present on the surface of traditionally dyed silk fabric.

## 4. Conclusions

For a better understanding of Korean plant-extracted dyes and traditionally dyed textiles, the application of complementary noninvasive and micro-destructive surface techniques were established. Reference silk textiles were prepared with commercial natural dyes and were characterized by FTIR, XPS and TOF-SIMS. The analysis of silk fabric dyed with natural extracts from plants by traditional dyeing processes proved the usefulness of the reference data, which were compiled from the results of systematic testing on dyed silk fabric with commercial natural dyes. The FTIR technique did enable individual dye components of the dyeing materials in silk fabric to be distinguished, thus providing less possibility to discriminate between dyes belonging to the same chemical class and, therefore, not giving comprehensive information about the analyzed silk fabric. However, this technique can be well exploited as a preliminary scanning prior to sampling in order to minimize the sampling number.

Micro-destructive surface techniques, such as XPS and TOF-SIMS, led to the acquisition of detailed information on natural dyes, both in the reference silk fabric with commercial dyes and in silk textiles dyed with natural plant extracts by traditional process. The major advantages of XPS and TOF-SIMS to characterize the natural dyes and traditionally dyed textiles included small sample size requirements, minimal sample preparation, the lack of chemical or mechanical pretreatments and identification of the molecular structures of dye components. Quantitative results for various mordants in sappanwood-dyed silk fabric and for oxygen containing functional groups in traditionally dyed silk fabric were obtained by XPS technique. TOF-SIMS spectra of silk fabric dyed with various plant extracts were obtained in the mass range of m/z 0–500. Molecular ions and fragment ions from the plant-extracted dyes of traditionally dyed silk fabric were observed, as were elemental ions from the mordants. Establishment of surface analytical methods and systematic accumulation of reference data will provide a good understanding of traditional dyeing process in Korea.
